# Efficiency of Points of Dispensing for Influenza A(H1N1)pdm09 Vaccination, Los Angeles County, California, USA, 2009

**DOI:** 10.3201/eid2004.130725

**Published:** 2014-04

**Authors:** Shubhayu Saha, Brandon Dean, Steven Teutsch, Rebekah H. Borse, Martin I. Meltzer, DeeAnn Bagwell, Alonzo Plough, Jonathan Fielding

**Affiliations:** Centers for Disease Control and Prevention, Atlanta, Georgia, USA (S. Saha, R.H. Borse, M.I. Meltzer);; Los Angeles County Department of Public Health, Los Angeles, California, USA (B. Dean, S. Teutsch, D. Bagwell, A. Plough, J. Fielding)

**Keywords:** H1N1, mass vaccination, points of dispensing, throughput, access, influenza, pandemic, influenza A(H1N1)pdm09, pH1N1, vaccination, vaccine, immunization, California, United States

## Abstract

During October 23–December 8, 2009, the Los Angeles County Department of Public Health used points of dispensing (PODs) to improve access to and increase the number of vaccinations against influenza A(H1N1)pdm09. We assessed the efficiency of these units and access to vaccines among ethnic groups. An average of 251 persons per hour (SE 65) were vaccinated at the PODs; a 10% increase in use of live-attenuated monovalent vaccines reduced that rate by 23 persons per hour (SE 7). Vaccination rates were highest for Asians (257/10,000 persons), followed by Hispanics (114/10,000), whites (75/100,000), and African Americans (37/10,000). Average distance traveled to a POD was highest for whites (6.6 miles; SD 6.5) and lowest for Hispanics (4.7 miles; SD ±5.3). Placing PODs in areas of high population density could be an effective strategy to reach large numbers of persons for mass vaccination, but additional PODs may be needed to improve coverage for specific populations.

Mass vaccination outside clinical settings (e.g., in pharmacies, workplaces, businesses, schools, and religious institutions) has been used to safely and efficiently provide a high volume of influenza vaccinations (*1*) and expand access to the vaccine (*2**,**3*). Success for such operations depends on the rapid dispensation of vaccines, the number of vaccines administered, and the communities reached. In Los Angeles County, California, USA, the 2009 influenza A(H1N1) pandemic was considered widespread by September 20, 2009 (*4*,*5*). Distributing the influenza A(H1N1)pdm09 (pH1N1) vaccine through points of dispensing (PODs) was the principal prevention strategy of the Los Angeles County Department of Public Health (LACDPH). PODs are vaccination clinics that operate at designated locations throughout the community for the temporary, large-scale dispensing of vaccines to persons at risk during a public health emergency. During October 23–December 8, 2009, the LACDPH distributed the pH1N1 vaccine through 60 POD locations in Los Angeles County. Clinical and nonclinical staff (including LACDPH-trained volunteers) registered patients and facilitated the vaccination process. The PODs were placed throughout the county to reach diverse, high-risk populations who would be less likely to receive the vaccine otherwise (*6*). We reviewed data from this effort to determine how future mass vaccination campaigns can improve the efficiency of vaccination at PODs and provide equitable access to PODs among demographic groups considered especially vulnerable to the vaccine-preventable outcome.

## Methods

We combined data collected at the PODs with census tract–level demographic information for Los Angeles County. We used a combination of multivariate regression analysis and geospatial methods to determine what factors affected the rate of vaccination (throughput) in the PODs; if the distance to PODs was similar for the 4 major ethnic groups living in Los Angeles County (white, African American, Hispanic, and Asian); how proximity to PODs affected visit patterns across these ethnic groups; and how the rate of POD visits varied by the underlying ethnic concentration and income status among the census tracts in Los Angeles County.

To examine throughput, we used data from each of the 101 POD events (some of the 60 locations had POD events on >1 day). Trained personnel completed patient registration forms that contained information about age, sex, ethnicity, address, existing medical conditions, pregnancy, and the type of pH1N1 vaccine received (i.e., live-attenuated monovalent vaccine [LAMV] administered nasally or monovalent inactivated vaccine injected from a multidose vial or as a single-dose unit). The number of hours staff worked was recorded, and each staff member was classified as clinical or nonclinical. We analyzed vaccine throughput (the average number of doses of vaccine administered per hour per POD event [dependent variable]) as a linear function of the following: vaccine mix (percentage of LAMV administered at each POD event); clinical staff time (percentage of hours worked by clinical staff/POD event); queue length (average number of persons waiting in line at each POD event); PODs same day (the number of PODs that were in operation in separate locations on the same day); high-risk patients (percentage of patients who were <10 years of age, were pregnant, or reported high-risk medical conditions and vaccine-related contraindications); previous influenza vaccination clinic held at site (previous seasonal influenza vaccination held at the same POD location); and vaccine shortage (POD operating on a day during the reported shortage of vaccine supply, which ended November 21).

We found the vaccine throughput data to be normally distributed by using the Shapiro-Wilk statistic (*7*), providing justification for using least squares regression (*8*). Because some locations had POD events on multiple days, we tested for systematic patterns of vaccination throughput by using White test for heteroscedasticity. Such patterns existed, and we corrected by estimating robust standard errors in which we used PODs as the clustering variable (*8*). A Ramsey test confirmed that no combination of higher order terms of the explanatory variables would fit the data better (*9*). Because estimates of the average number of persons waiting outside the POD were skewed, we log transformed the data measuring queue length. We used Stata version 10 (StataCorp, College Station, Texas, USA) for the regression analysis.

The datasets used for the spatial analyses were population-weighted geometric center of each census tract from the 2000 census, census tract–level distribution of ethnic populations and median household income in 2009, geocoded addresses of patients obtained from patient registration forms, and geocoded POD locations. We used Centrus software (Stamford, CT, USA) for geocoding and ARCGIS version 9.2 software (ESRI, Redlands, CA, USA) for spatial analyses. For each patient, we geocoded the residence address, identified the census tract containing the geocoded address, and calculated the Euclidean (straight-line) distance between the geocoded address and each of the POD locations. We also calculated the Euclidean distance between each population-weighted census tract geographic center and each of the POD locations. We used census tract–level demographic data to classify each census tract into ethnic population quartiles (white, African American, Hispanic, and Asian). Each census tract was also assigned to an income quartile on the basis of the tract’s median household income. Thus, each of the 2,052 census tracts in Los Angeles County was classified into 4 ethnic quartiles and 1 income quartile.

We first assessed the average distance to PODs for members belonging to the 4 ethnic groups. For each census tract, we identified the POD closest to the tract geographic center and assumed that all persons living in the census tract traveled the same distance to reach that closest POD. We then used the number of persons in each ethnic group in each census tract as weights to calculate a weighted average distance to the nearest POD for each ethnic group. For sensitivity analysis, we repeated the process to calculate the average distance to the second and third closest POD.

We then assessed how proximity to PODs affected visit patterns to PODs across ethnic groups. We calculated the percentage of patients in the 4 ethnic groups who visited any of the 3 PODs closest to the census tract where they lived by using the census tract in which the geocoded address of a patient fell and geocoded address of the POD that each patient visited. For each ethnic group, we then calculated the percentage of patients who visited any of the 3 PODs closest to them.

To ensure that certain subpopulations (e.g., low income, ethnic minorities) have adequate access to PODs, the units are often located in areas with a high density of those subpopulations to achieve high rates of vaccination. Thus, we assessed how frequency and rates of POD visits varied by the distribution of ethnic population and median household income. We used the geocoded patient address data to identify the number of patients in each ethnic group residing in each census tract. For each census tract, we then calculated the number of patients by each ethnic group. Using this count as the numerator and the total population for each ethnic group as the denominator, we calculated an ethnicity-specific POD visit rate for each census tract. We summarized these numbers across census tracts by ethnic population and income quartile to calculate count and average visit rates by ethnic groups.

## Results

Descriptive statistics of the variables used in the regression analysis of vaccine throughput during the 101 POD events are shown in Table 1. A total of 179,688 vaccine doses were administered at the PODs; the average number of doses administered per hour per POD was 239 (range 40–427). LAMV constituted an average of 29% of the vaccines administered at a POD (range 0%–62%). Clinical staff contributed an average of 56% (range 12%–100%) of total staff time, which included hours worked by nonclinical staff and volunteers. Approximately 6 (range 1–13) POD events were held per day, but delays in vaccine availability caused variation over time. pH1N1 vaccines were in short supply until November 21, 2009, and ≈81% of the PODs were in operation before that date. Approximately 14% of POD locations had previously been used for seasonal influenza vaccinations. An average of 247 persons (range 7–1,614) every hour were estimated to be in line outside a POD waiting to be vaccinated. Of all patients, 22% were 0–10 years of age, 4% were pregnant, and 23% reported medical conditions that influenced the type of vaccine they received.

**Table 1:** Patient characteristics and POD data from 101 influenza vaccine events, Los Angeles County, California, USA, October 23–December 8, 2009*

Variable	Mean	SD	Min	Max
Patient characteristic, % patients				
Age 0–10 y	22	7	0	41
Pregnancy	4	3	0	25
Contraindications	3	7	0	42
Preexisting medical conditions	23	7	0	36
POD data				
Throughput	239	87	40	427
Vaccine mix	29	13	0	62
Clinical staff time	56	13	12	100
Queue length	247	277	7	1,614
PODs same day	5.7	3.7	1	13
Proportion of POD locations with influenza vaccine clinics before 2009	0.14	0.08	0	1
Proportion of POD events during vaccine shortage ending on 2009 Nov 21	0.81	0.15	0	1

*POD, point of distribution; throughput, average vaccine doses administered per hour per POD event; vaccine mix, percentage of live-attenuated monovalent vaccine; clinical staff time, percentage of clinical staff hours; queue length, average number of patients in queue outside PODs per hour per event; PODs same day, number of POD events held on the same day.

The baseline rate of vaccination was ≈251 patients per hour (SE 65; p<0.01) (Table 2). A 10% increase in LAMV in the vaccine mix was associated with a 23.2% decrease in throughput (SE 0.7; p<0.01) (Table 2). A similar increase in the percentage of clinical staff hours was associated with an 11% decrease in persons vaccinated per hour (SE 0.61; p = 0.06). A 10% increase in the average number of persons waiting in line outside a POD was associated with an increase in throughput of 3 patients per hour (SE 6.88; p<0.01). Operation of other PODs on the same day at another location, percentage of patients who were 0–10 years of age, and percentage of patients who reported contraindications and preexisting medical conditions did not significantly affect vaccine throughput.

**Table 2:** Factors affecting average number of patients vaccinated per hour (throughput) per influenza vaccine POD event, Los Angeles County, California, USA, October 23–December 8, 2009*†‡

Variable†	No. persons/h (95% CI)	SE‡	p value
Baseline	251.19 (124.43 to 377.95)	64.68	0.00
Patient demographic			
Age 0–9 y	–2.54 (–5.87 to 0.79)	1.70	0.14
Pregnancy	4.55 (–0.78 to 9.88)	2.72	0.09
Contraindications	–2.53 (–6.14 to 1.09)	1.85	0.17
Preexisting medical conditions	1.38 (–1.69 to 4.44)	1.56	0.38
POD data			
Vaccine mix	–2.32 (–3.69 to –0.96)	0.70	0.00
Clinical staff time	–1.14 (–2.33 to 0.06)	0.61	0.06
Queue length	32.05 (18.57 to 45.53)	6.88	0.00
PODs same day	–3.21 (–8.06 to 1.63)	2.47	0.19
Influenza clinic in same location before 2009	37.32 (–13.78 to 88.43)	26.07	0.15
Period of vaccine shortage	–17.56 (–64.43 to 29.31)	23.92	0.46

*N = 101. Adjusted R^2^ = 0.57; Wald χ^2^ = 144.6 (Prob > χ^2^ = 0.00). POD, point of distribution; throughput, average vaccine doses administered per hour per POD event; vaccine mix, percentage of live-attenuated monovalent vaccine (LAMV); clinical staff time, percentage of clinical staff hours; queue length, average number of patients in queue outside PODs per hour per event; PODs same day, number of POD events held on the same day. †The analysis estimates the effect of these variables on the baseline number of persons vaccinated per hour per individual POD. For example, 1% increase in LAMV reduced throughput by 2.32 persons vaccinated per hour. ‡Robust clustered SEs with POD as clustering variable.

The largest ethnic group in Los Angeles County is Hispanic (47% of the population); a similar percentage (44%) for this group was found among those came to PODs for vaccination (Table 3). The rate of vaccination for the total population across all PODs was highest for Asians (257/10,000 persons), followed by Hispanics (114/10,000 persons), whites (75/10,000 persons), and African Americans (37/10,000 persons).

**Table 3:** Residence, rate of vaccination, and distance traveled for persons visiting PODs for influenza vaccination, Los Angeles County, California, USA, October 23–December 8, 2009*

Variable	White	African American	Hispanic	Asian
% Residents of Los Angeles†	30	9	47	13
Total % patients in PODs,‡ n = 125,849	19	3	44	28
Number vaccinated per 10,000 population	75	37	114	257
Average distance traveled, miles (SD)§	6.6 (6.5)	5.6 (6.3)	4.7 (5.3)	6.3 (6.2)
Median distance traveled, miles§	4.7	3.8	2.9	4.5

*PODs, points of distribution. †2009 tract-level demographic data from the Los Angeles Department of Public Health. ‡Information from questionnaire used at POD before vaccination. §Euclidean distance from residence to POD based on geocoded addresses of vaccines.

Of persons who received the 179,688 vaccine doses administered in the PODs, 157,176 were residents of Los Angeles County. A total of 125,849 addresses provided at the POD registration could be geocoded; spatial analyses were restricted to this sample. On the basis of the calculated Euclidean distance between geocoded residence and PODs, the average distance traveled to a POD was highest for whites (6.6 miles; SD 6.5) and lowest for Hispanics (4.7 miles; SD 5.3). The average distance to the closest POD was relatively the same across ethnic groups: 2.7 miles for whites, 2.2 miles for Asians, and 2.0 miles for African Americans and Hispanics (Table 4). The average distances to the second and third closest PODs were lowest for African Americans and highest for whites. Hispanics were most likely to visit the POD closest to the geographic center of the census tract where they lived (44%), followed by whites (39%), African Americans (35%), and Asians (31%) (Table 4). By adding the percentages of patients who visited the 3 closest PODs, we found that >50% of patients in each ethnic group attended 1 of the 3 PODs closest to where they lived (Table 4).

**Table 4:** Average distance persons traveled to the 3 closest PODs for influenza vaccination, Los Angeles County, California, USA, October 23–December 8, 2009*

POD location	Distance traveled to POD, miles (% persons visiting POD)			
	White	African American	Hispanic	Asian
Closest	2.7 (39)	2.0 (35)	2.0 (44)	2.2 (31)
2nd closest	5.0 (15)	3.4 (12)	3.5 (12)	3.8 (13)
3rd closest	6.3 (8)	4.4 (8)	4.4 (9)	4.8 (10)

*Values do not equal 100% for each category because some patients visited PODs not among the 3 closest to where they live. PODs, points of distribution.

Of patients who attended a POD, 84% resided in census tracts in the third and fourth quartiles for population density of the associated ethnic group. This finding indicates that placing PODs in closer proximity to high population density centers could increase the total number of persons vaccinated. However, these patterns are less apparent when rates of POD visits are examined. For example, the rate of POD visits for Asians (267/10,000), Hispanics (132/10,000), and African Americans (83/10,000) were highest from the census tracts in the bottom of their respective population quartiles (Table 5). Differences between the rates across the population quartiles did not vary by ethnic group, except for African Americans, for whom the average rate for the bottom 2 quartiles was more than double that of the average rate for the top 2 quartiles (Table 5).

**Table 5:** Numbers and rates of persons receiving influenza vaccine at PODs by ethnic group and population density, Los Angeles County, October 23–December 8, 2009*

Population density quartile	No. persons (rate†)			
	White	African American	Hispanic	Asian
Bottom	646 (74)	353 (83)	2,682 (132)	1,835 (267)
Second	2,033 (58)	392 (60)	7,362 (109)	3,314 (221)
Third	6,191 (70)	572 (36)	16,385 (114)	7,053 (239)
Top	14,606 (81)	2,157 (32)	29,567 (114)	22,832 (266)

*Quartiles based on ethnic population distribution across census tracts. POD, point of distribution †No./10,000 population for the ethnic group.

POD visit patterns differed across ethnicities based on median household income in census tracts. A total of 55% of African American and 68% of Hispanic POD attendees came from census tracts in the lowest 2 income quartiles. This percentage was much smaller for whites (18%) and Asians (36%) (Table 6). However, the rate of POD visits uniformly increased from the lowest income quartile to the highest income quartile (Table 6). The difference in the rate across the income quartiles was most apparent among African Americans, from 28/10,000 for the lowest to 71/10,000 for the highest income quartile (Table 6).

**Table 6:** Numbers and rates of persons receiving influenza vaccine at PODs by ethnic group and median household income, Los Angeles County, California, USA, October 23–December 8, 2009*

Income quartile	No. persons (rate†)			
	White	African American	Hispanic	Asian
Bottom	1,245 (54)	1,013 (28)	18,032 (103)	4,825 (227)
Second	3,058 (57)	909 (31)	19,878 (116)	7,866 (238)
Third	6,771 (67)	846 (46)	13,324 (124)	11,728 (270)
Top	12,402 (91)	706 (71)	4,762 (126)	10,615 (274)

*Quartiles based on median household income distribution across census tracts. POD, point of distribution †No./10,000 population for the ethnic group.

## Discussion

We retrospectively evaluated pH1N1 vaccine distribution through PODs in Los Angeles County to identify factors associated with throughput of patients and vaccine coverage for different racial/ethnic groups. We found that a higher proportion of LAMV among vaccines administered and higher proportion of clinical staff among all personnel were associated with a reduction in throughput of vaccine. An increase in the proportion of pregnant woman among patients was associated with increased throughput; anecdotal evidence suggests the reason may be that pregnant women were provided a dedicated, priority queue at the PODs, and only 1 type of vaccine was used.

The rate of vaccination in the PODs was highest for the Asian residents of Los Angeles County, followed by Hispanics, whites, and African Americans. PODs were placed throughout Los Angeles County equally close to different racial/ethnic groups. Across all 4 ethnic groups, >80% patients resided in top 2 population density quartiles of their respective ethnicities. Thus, placing PODs in census tracts of high population density could be an effective strategy to reach large numbers of persons. Income quartile notably affected rates of vaccination for whites and African Americans but had very little effect for Hispanics and Asians.

Our findings add to the understanding of the association between POD-level features and vaccination rates and helps elucidate the usefulness of geographic information systems in planning improved community-level access to health care (*10**,**11*). Spatially indexed clinic-level health data aligned with commonly available census information have been used to identify patient catchment areas and assess underserved populations (*12*). Spatial statistics has been used to identify patterns in use of psychiatric outpatient care among racial and ethnic groups to inform the design of interventions to improve access to care (*13*). Wheras access to care in this study was measured on the basis of Euclidean distances between POD location and patient addresses (specific travel information to PODs for each patient was not available), actual travel route and time may be more informative, but reports in the literature do not agree on this point (*13**,**14*).

Our study has several limitations. Most of the information used in the analyses was recorded in paper-based questionnaires before vaccines were administered at the PODs. Use of modern technology (e.g., electronic data recorders) at times of such emergencies could possibly reduce the rate of missing and incomplete information. Our analysis further did not account for the differential effectiveness of crowd-management techniques (e.g., dedicated lines for certain persons) that could facilitate faster movement of patients through the PODs. Such information was not available for all PODs. The patterns in POD visits we found for Los Angeles County depended on the underlying spatial distribution of different ethnic populations, which also limits the generalizability of the patterns we would find for other jurisdictions. Patterns of POD visits during the 2009–10 pandemic were likely influenced by a wide variety of unmeasured factors (e.g., perceived severity, availability of vaccine relative to timing of pandemic). Although we controlled for some of these supply issues in the regression analysis, we did not have the necessary information to take these factors into account for the spatial analyses.

In a community without spatially pronounced racial and ethnic or socioeconomic population distributions, selecting POD sites on the basis of population densities may prove the most efficient strategy. Such a method may ensure the greatest possible community-wide coverage during a pandemic. However, ensuring equitable reach to all ethnic subgroups may require the use of more strategies designed to target select subpopulations, particularly for a community with marked clustering of definitive population subgroups. Similar analyses should be conducted in other locations to inform public health preparedness activities in similar future scenarios (*15*).

Making comprehensive policy recommendations for emergency public health operations is a challenge because all emergencies are local and driven by factors unique to each emergency and location (*16*). However, this vaccination campaign represents one of the largest POD-based efforts in the history of emergency public health response, and several approaches could be used to improve outcomes in future public health emergencies. Limiting the variety of available medical countermeasure products (i.e., different vaccine types, multiple antimicrobial drugs) at PODs could reduce the amount of time and resources needed to triage and match clients to a particular product. In addition, optimizing the ratio of clinical staff to nonclinical staff would maximize efficiency while ensuring a safe system. Furthermore, placing PODs close to population clusters would serve the dual objectives of wide coverage and representation of population subgroups (*17*).
